# Left ventricular inferior wall congenital diverticula in athletes: a case series and review of the literature

**DOI:** 10.1093/ehjcr/ytae036

**Published:** 2024-01-23

**Authors:** Giuseppe Di Gioia, Lorenzo Buzzelli, Andrea Segreti

**Affiliations:** Institute of Sport Medicine and Science, National Italian Olympic Committee, Largo Piero Gabrielli, 1–00197 Rome, Italy; Department of Cardiovascular Sciences, Fondazione Policlinico Campus Bio-Medico, Via Alvaro del Portillo, 200–00128 Rome, Italy; Department of Movement, Human and Health Sciences, University of Rome ‘Foro Italico’, Piazza Lauro De Bosis, 15–00135 Rome, Italy; Department of Cardiovascular Sciences, Fondazione Policlinico Campus Bio-Medico, Via Alvaro del Portillo, 200–00128 Rome, Italy; Department of Cardiovascular Sciences, Fondazione Policlinico Campus Bio-Medico, Via Alvaro del Portillo, 200–00128 Rome, Italy; Department of Movement, Human and Health Sciences, University of Rome ‘Foro Italico’, Piazza Lauro De Bosis, 15–00135 Rome, Italy

**Keywords:** Congenital aneurysms, Congenital diverticula, Echocardiography, Cardiac magnetic resonance imaging, Case series, Athletes

## Abstract

**Background:**

Congenital left ventricular diverticula (LVDs) and aneurysms (LVAs) are rare, developmental, cardiac anomalies, which are often asymptomatic. Sometimes they can cause life-threatening complications like arrhythmias, syncope, embolic events, ventricular wall rupture, valvular regurgitation, congestive heart failure, and various symptoms. Diagnosis is usually made after exclusion of acquired causes, from cardiac or non-cardiac disorders. Specific guidelines for LVD/LVA management are not available and treatment options are guided by different case-by-case clinical presentation and possible complications.

**Case summary:**

We present a series of two patients with occasional diagnosis of diverticula of the inferior basal left ventricular wall in the context of cardiological evaluations for competitive sport certificate. Symptoms were present at clinical evaluation only in Patient 1, together with electrocardiogram (ECG) abnormality. We performed transthoracic echocardiography as a first-line examination and secondly, we confirmed the diverticula by cardiac magnetic resonance. A maximal stress test and 24 h ECG Holter were also performed.

In our case, in light of the clinical-instrumental findings, periodic medical and echocardiographic follow-up without therapy was established, together with the resumption of sports activities.

**Discussion:**

Nowadays, no specific recommendations exist in athletes and no studies are available on how regular sport practice can influence natural history of LVD/LVA. The current case series highlights the importance of risk stratification for cardiac events, of a multimodal imaging approach in diagnostic procedure and of a tailored treatment strategy.

**Learning points**

- Congenital left ventricular diverticula and aneurysms are rare cardiac anomalies, which are often asymptomatic but sometimes they can cause life-threatening complications.
- Specific guidelines for management, both in the general population and in athletes, are not available and treatment options are guided by different case-by-case clinical presentation.
- It is recommended to carry out a risk stratification that directs the treatment to be followed.

## Introduction

Congenital left ventricular diverticula (LVDs) and aneurysms (LVAs) are rare cardiac anomalies, supposedly arising from a partial interruption of embryonic ventricle growth.^1^ Progresses in transthoracic echocardiography (TTE) and cardiac magnetic resonance (CMR) imaging^2,3^ allowed an earlier identification, including prenatal diagnosis.^4^ Nowadays prospective studies on epidemiology are lacking and no uniform classification with therapeutic/prognostic implications is available.^5,6^ Congenital left ventricular diverticulum is defined as a sac-like appendix consisting of all the three layers of heart’s wall (endocardium, myocardium, and epicardium) emerging from the left ventricle’s wall (LV), usually with a narrow connection to the ventricular cavity and a normal contractility.^1^ In opposition, LVA has a wide connection to the LV, composed mainly by connective tissue and, according to the amount of myocardial fibres involved, it can have normal contractility, a- or dyskinesia.^1^ Until 2012, 809 cases of congenital LVD/LVA are reported in literature: they represent distinct malformations, with different clinical manifestations, associated anomalies, histological characteristics, and prognosis.^2^ Most of LVA were localized in the inferior wall, in the perivalvular area (close to the mitral valve), and at LV apex.^2,7^ Instead, the clear majority of the LVD is found at the LV apex.^2,7^ LVD/LVA can be associated with numerous additional congenital cardiac, vascular, and extracardiac anomalies, but LVA is more frequently isolated and prevalence of associated defects is significantly higher in LVD.^2,7^ A small retrospective study showed a higher prevalence of coronary anomalies in patients with isolated LVA and LVD, specifically in males and in patients with non-apical LVA or LVD.^8^ Clinically, great majority of LVA/LVD is often asymptomatic and usually found incidentally during diagnostic procedures executed for other reasons.^7^ In symptomatic patients, arrhythmias and syncope, embolic events, LVA/LVD rupture, and congestive heart failure (CHF) were the main clinical manifestations.^7^ Ventricular arrhythmic events, with morphologies corresponding to anatomical location of the lesions and reproducible inducibility during electrophysiological studies, were significantly higher in patients with LVA compared to LVD, with no differences in the incidence of syncope.^2,7^ Cardio-embolic events were generally uncommon at presentation, but description of thrombotic material inside the anomaly was significantly more frequent in LVA patients.^7^ Rupture as the cause of presentation was mostly reported in younger patients, particularly in the perinatal period, with no differences between LVA and LVD.^7^ Congestive heart failure was significantly more frequent in LVA patients: in prenatal and young patients, a large aneurysm can lead to a poorly tolerated volume overload in the foetal or neonatal heart, while in older age patients, close anatomical proximity to the valve structures (sub-aortic or sub-mitral LVA/LVD) can cause regurgitation of the aortic or mitral valve secondary to valve’s annulus distortion.^7^ Typical angina or atypical chest pain was more frequently observed in LVD patients.^7^ Diagnosis requires exclusion of coronary artery disease (main cause of acquired aneurysms), local or systemic inflammation, traumatic causes, non-cardiac or systemic diseases, and cardiomyopathies.^1,2,7^ Occasionally, chest radiography can help to observe alterations of the cardiac silhouette and radionuclide studies can estimate the amount of viable myocardium in the wall of the LVA/LVD.^7^ Electrocardiogram (ECG) is not able to diagnose accurately congenital LVA or LVD, but Ohlow *et al*.^9,10^ showed an higher incidence of distinctly abnormal ECGs in older patients with an apical location of the anomaly. Moreover, the presence of these ‘distinct’ ECG alterations is relevant for the prognosis, as the incidence of clinical events at follow-up was significantly higher in these patients and in those with symptoms.^9,10^ Transthoracic echocardiography is a universally available non-invasive tool and represents the first-line diagnostic technique.^7^ Cardiac magnetic resonance is the gold-standard imaging method for quantification of ventricular volumes, mass, and ejection fraction (EF), providing both anatomic and functional information together with tissue characterization.^3,11^ It can also visualize any associated complex congenital cardiac anomalies and, through MR-angiography, the blood flow to and from LVD or LVA.^2^ Specific guidelines for LVA/LVD management are not available. Treatment options (mainly surgical vs. observational follow-up) are directed by clinical presentation and findings of the individual patient. Surgical technique consists in LVD/LVA resection (aneurysmectomy, direct suturing of LVD orifice or resection with patch closure) and it depends on the type and extension of the lesion.^7^ Non-surgical strategy with careful follow-up has been chosen in many case reports and it demonstrated an overall benign course with an event rate of ∼1.2% per year.^7,12,13^ In another small series of neonatal/juvenile patients, the event rate was higher (6% per year), suggesting an interventional therapy in this subgroup of patients.^14^ Currently, available data about outcomes are limited and mainly can be extrapolated from a systematic analysis of about 800 LVA/LVD published in the last two centuries.^2^ Congenital left ventricular aneurysm patients showed more adverse cardiac events [arrhythmic events, rupture, sudden cardiac death (SCD), CHF, embolic events, syncope] during follow-up, especially younger patients.^2^ Generally, symptoms at presentation and several distinct ECG abnormalities have turned out to be possible predictive factors about the incidence of adverse events.^2^ In a large single-centre retrospective study, one-third of the LVA/LVD patients had cardiovascular events at follow-up, with a prevalence of rhythm disturbances in LVA group and a higher incidence of embolic events in LVD group.^15^

## Summary figure

**Table:** 

	Age, gender	Symptoms	ECG	Maximal stress test and 24 h ECG Holter monitoring	TT echo	CMR	Management
Case 1	23, F	Shortness of breath, tachycardia (with slow return to basal heart rate after mild effort) and dizziness after moderate effort	Presence of significant Q waves, not regressed with deep inspiration, in inferior leads	Within the norm	LV of normal dimension and wall thickness. Mild mitral regurgitation; EF 65%. Giant diverticulum of the inferior basal wall with thinning of the underlying wall but with preserved motion.	Confirmation of the diverticulum of basal inferior wall. Normal contractility. No late-gadolinium enhancement (LGE)	Periodic medical and echocardiographic follow-up without therapy, with resumption of sports activities
Case 2	42, M	Asymptomatic	Mild and non-specific ventricular abnormalities in inferior leads	Within the norm	EF 60%. Trivial mitral regurgitation. Evidence of a diverticulum of basal inferior wall.	Confirmation of the diverticulum of basal inferior wall. Normal contractility. No LGE	Periodic medical and echocardiographic follow-up without therapy, with resumption of sports activities

Here, we report a case series of two athletic patients with LVD of inferior wall. Informed written consent was obtained from the subjects.

## Patient 1

G, 23 years old, female came to our attention for cardiological evaluation for competitive sport certificate. She practiced tennis since the age of 8 years and started to require competitive sport certification in the last three years. Previous sport medicine evaluations did not request further cardiological exams and she always obtained competitive certification. She participated in local and regional competitions, with a volume of training of 7 h per week characterized by 5 of tennis and 2 h of isometric training.

**Figure 1:**
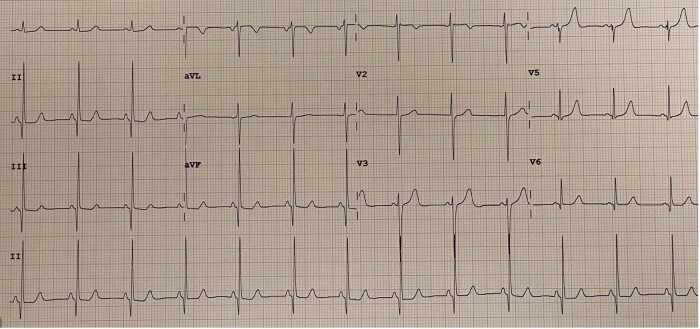
Patient 1, rest ECG showing inferior Q waves.

**Figure 2:**
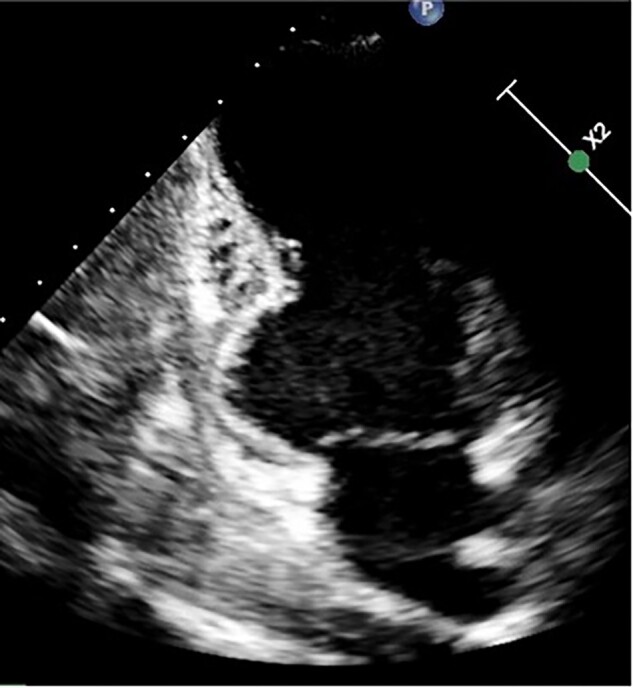
Patient 1, transthoracic echocardiogram: apical two-chamber view showing diverticulum of the inferior basal wall of the left ventricle (length 3.4 cm, depth 2.4 cm).

**Figure 3:**
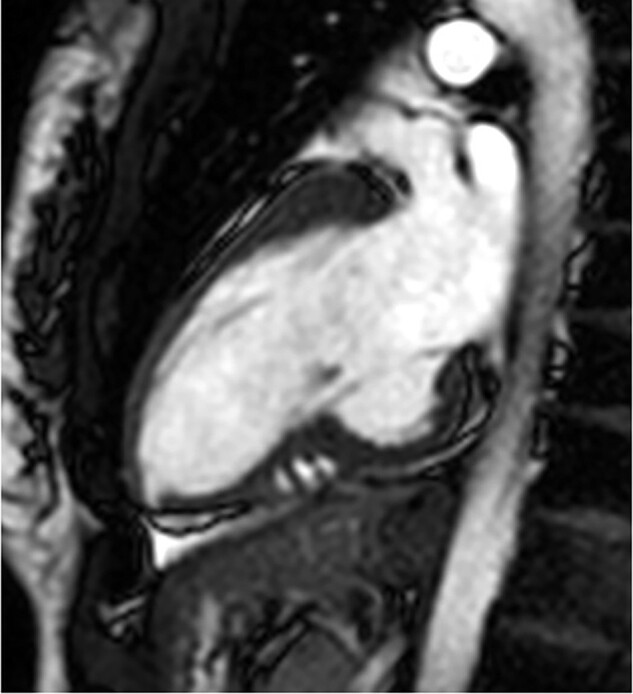
Patient 1, cardiac magnetic resonance: two-chamber view confirmed giant diverticulum of the inferior basal wall.

Recently, the patient referred onset of symptoms characterized by shortness of breath, tachycardia (with slow return to basal heart rate after mild effort), and dizziness after moderate effort. Her cardiovascular history was negative; she had normal body mass index (20,7), never smoked, and denied any previous disease or surgical procedures; no cardiovascular risk factors were evident. She did not take pharmacological therapy. In her family, maternal grandmother had hypertrophic cardiomyopathy and grandmother’s brother died at 51 years old for SCD. Two daughters of the dead uncle, nowadays 60 and 63 years old, have hypertrophic cardiomyopathy [one with implantable cardioverter defibrillator (ICD) implantation]. She denied SARS-COV2 infection, submitted to three doses of Comirnaty vaccine. Recent blood tests (three weeks earlier than our evaluation) including full blood count, renal and liver function, electrolytes, and glucose were normal. Cardiological evaluation showed normal heart rhythm, clear tones, systolic 2/6 L heart murmur at cardiac base. Left omeral blood pressure was 120/80 mmHg. Rest 12-lead ECG (*Figure 1*) showed regular sinus rhythm with 78 b.p.m., normal QRS axis, normal atrioventricular and intraventricular conduction, normal ventricular repolarization, and normal QTc interval. In inferior leads presence of significant Q waves, not regressed with deep inspiration. She was then submitted to TTE that showed LV of normal dimension and wall thickness with slight increase of LV outflow velocity, mild mitral regurgitation; EF was preserved (65%). It was evident with a giant diverticulum (length 3.4 cm, depth 2.4 cm) of the inferior basal wall (*Figure 2*, Supplementary material online, *Video S1*), with thinning of the underlying wall but with preserved motion. Cardiac magnetic resonance confirmed huge congenital diverticulum of basal inferior wall with similar dimensions compared to TTE (*Figure 3*, Supplementary material online, *Video S2*) with normal contractility. No late-gadolinium enhancement (LGE) was observed. It was then performed a maximal stress test and 24 h ECG Holter monitoring that showed no ventricular arrhythmias.

Finally, considering the normal ventricular repolarization and QRS voltage at rest ECG, the absence of ventricular arrhythmias at exercise stress test and 24 h ECG Holter monitoring, the normal LV wall thickness at TTE and CMR and the absence of LGE, we decided to issue the competitive sport certificate and allow the patient to return to sport activity and we did not submit the patient to genetic screening. In fact, according to Italian Cardiological Guidelines (COCIS) for Competitive Sport Eligibility^16^ genetic screening is not indicated for screening in absence of phenotypical expression of LV hypertrophy.

The athlete returned to play tennis and denied any of the symptoms previously described. Considering the congenital diverticulum and the familiarity for hypertrophic cardiomyopathy, an annual echocardiographic follow-up was requested in order to evaluate early morphological changes suitable for hypertrophic cardiomyopathy.

## Patient 2

M, 42 years old, male, fitness trainer came to our outpatient clinic requiring a competitive sport medical certificate for cross-fit. He practiced aerobic sport disciplines since the young age; during this period, he required for several times competitive sport certification for soccer, swimming, and athletics and he had negative history of sports disqualification. At the time of our evaluation, he trained for 10 h per week with 8 h of isometric exercise and the remaining with aerobic training.

**Figure 4:**
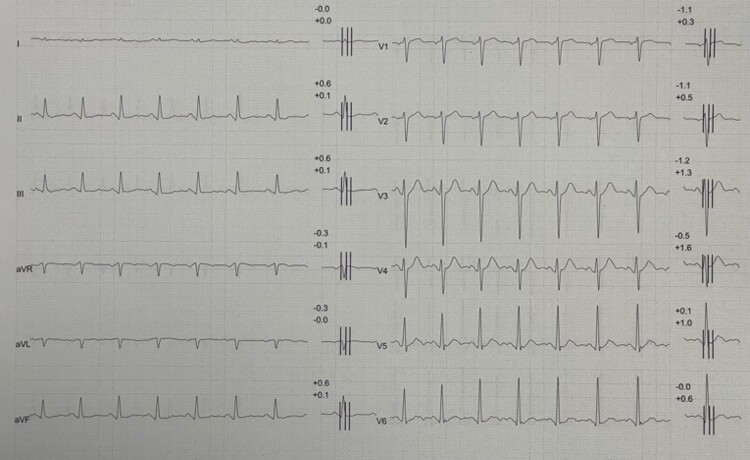
Patient 2, rest ECG showing mild and non-specific ventricular abnormalities in inferior leads.

**Figure 5:**
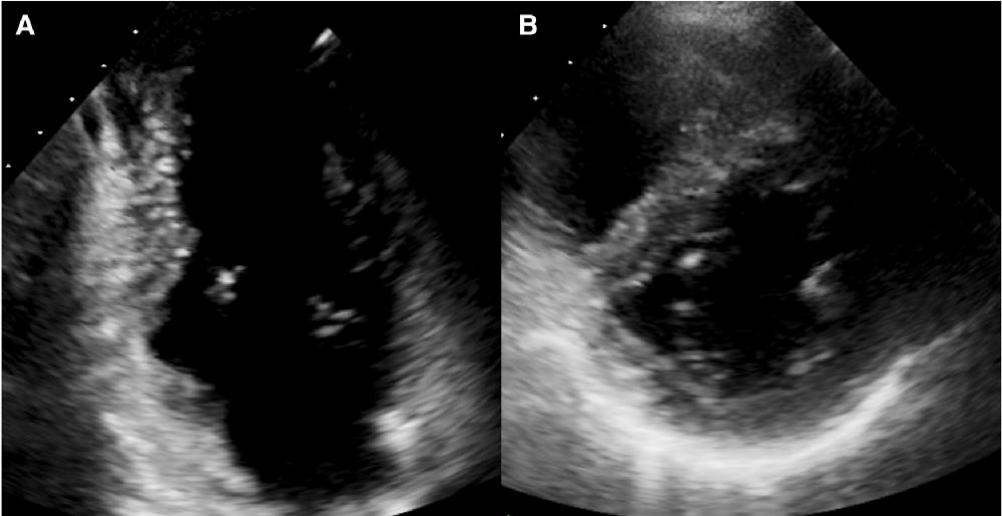
Patient 2, transthoracic echocardiogram: (*A*) apical two-chamber view showing diverticulum of the basal portion of inferior wall (length 3.0 cm, depth 1.9 cm). (*B*) Parasternal short axis view.

**Figure 6:**
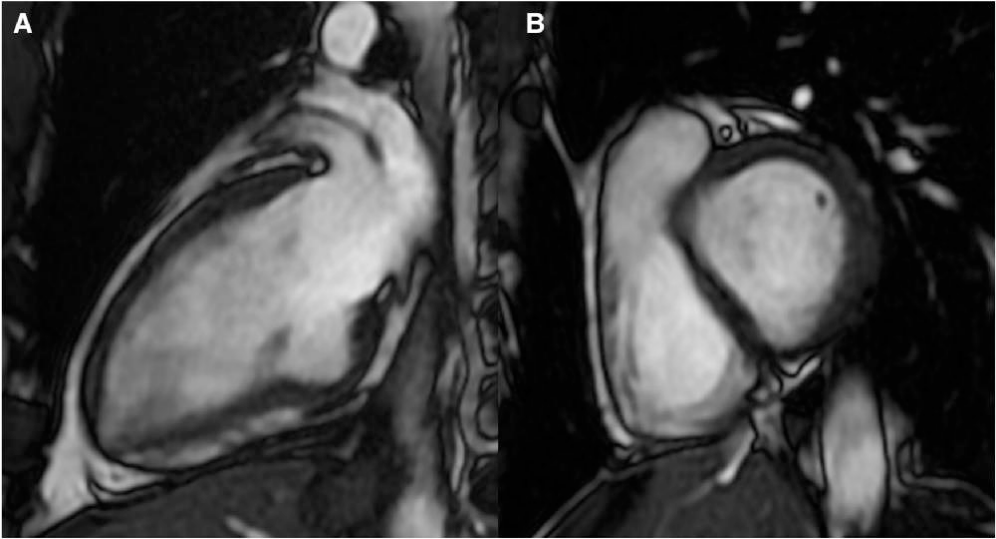
Patient 2, cardiac magnetic resonance. (*A*) Two-chamber view; (*B*) basal short axis view.

He was in good global health; denied systemic diseases or surgical procedures. His cardiological history was negative, without any cardiovascular risk factors. He was asymptomatic, regularly practicing aerobic and strength exercises without any symptom. No family history for cardiovascular disease was present. A rest ECG was performed (*Figure 4*) showing normal sinus rhythm with 90 b.p.m., normal QRS axis, normal atrioventricular and intraventricular conduction, normal ventricular repolarization, normal QTc interval, and mild and non-specific ventricular abnormalities in inferior leads. Stress test resulted negative for coronary reserve reduction without any significant ST-T modification at peak. A TTE was therefore performed (*Figure 5*) with evidence of a diverticulum of basal inferior wall (length 3.0 cm, depth 1.9 cm), with preserved EF (60%) and trivial mitral regurgitation. A 24 h ECG Holter was performed and resulted negative without ventricular arrhythmias. Therefore, CMR (*Figure 6*, Supplementary material online, *Video S3*) confirmed the presence of the diverticulum without any scar or LGE. Also in this case, a global clinical and instrumental morpho-functional evaluation was performed before to issue competitive sport certificate: the absence of symptoms and ventricular arrhythmic burden, the normal ECG, and the normal contractility of the diverticulum associated to the lack of LGE at CMR suggested the lack of indication for treatment; so sport activities were restarted and periodic annual echocardiographic follow-up requested.

The data underlying this article are available in the article and in its online Supplementary material.

## Discussion

We observed two cases of LVD with concrete dimensions and atypical locations, at basal portion of LV inferior wall in patients practicing competitive sport. Management of LVD/LVA is even more challenging in athletes, given the extremely low number of cases in the literature in this context, the absence of specific guidelines and the lack of data about outcome and how/if regular sport practice can influence natural history. Symptoms (shortness of breath, tachycardia, and dizziness) were present only in Patient 1, together with presence of significant Q waves in inferior leads. This ECG abnormality (Q waves 2 to 3 mm depth and in >2 leads) is among those described in a large single-centre study as ‘specific’ of LVA and LVD patients and has shown to be of prognostic importance.^9,10^ Cardiological guidelines for competitive sport eligibility^16^ recommend execution of rest ECG and exercise stress test as screening tools in athletes. However, a morphological evaluation is not required meaning that the diagnosis of LVA/LVD is mainly an incidental finding in TTE required for other reasons. Nevertheless, ECG is not specific and sensitive to be used as screening tool in such patients.^10^ Concurrently, although there are no specific recommendations regarding congenital aneurysms/diverticula, performing further structural investigations, such as TTE and eventually CMR, is reasonable in patients who report cardiovascular symptoms, in cases of even mild suspected ECG abnormalities, or in the presence of risk factors such as family history of heart disease. In this way, and in the specific case of such rare findings, considering together the elements derived from different diagnostic tools, cascading guided by clinical suspicion, leads to a comprehensive risk stratification. In addition, an adequate follow-up is appropriate to assess the temporal evolution of the aneurysms/diverticula, to better understand their clinical course and to periodically reassess eligibility for competitive sports, hopefully leading to new scientific evidence in the future to guide the management of these patients.

At the same time, Patient 1 reported a family history for hypertrophic cardiomyopathy and SCD. Congenital left ventricular diverticulum or LVA formation is also reported in different cardiomyopathies, including arrhythmogenic right ventricular cardiomyopathy with LV involvement^17^ and hypertrophic cardiomyopathy.^18–20^ In our case, multimodality imaging was crucial to outline the morphology. We performed TTE as a first-line examination and secondly, we confirmed the diverticula by CMR. Cardiac magnetic resonance is often recommended to discriminate LVD from LVA, to accurately define its location and dimensions and, finally, to assess ventricular wall composition, whether muscular or fibrous.^3,11^ In our cases, the location of the lesions was in contrast with the available data in literature, where most of the LVA are described to be localized in the inferior wall and in the sub-mitral area^7^ and the clear majority of the LVD is found at LV apex.^2,7^ There are very few cases reported in the literature of congenital LVD/LVA in athletes.^12,21,22^ After a thorough diagnostic investigation using the recommended techniques, conservative therapy was preferred in some of these,^12^ while in other cases,^21,22^ especially those presented with aborted sudden cardiac arrest or with CMR evidence of very thin and fibrous aneurysmal ventricular walls with LGE, interventional therapy such as ICD implantation or aneurysmectomy was preferred. In a case, after surgery, patient was recommended not to participate further in competitive sport.^20^ In our case, periodic medical and echocardiographic follow-up without therapy was established, together with the resumption of sports activities, as, in both cases, no arrhythmias were detected, good contractility and thickness of the diverticula with no LGE at CMR suggest a low probability of thrombus formation. However, a stricter and closer monitoring in Patient 1 is needed, on the basis of the presence of risk factors, as evidenced by previous studies in literature, for a possible increased incidence of clinical events: symptoms at onset, ECG abnormalities, and family history of SCD and hypertrophic cardiomyopathy.

## Conclusions

We presented two cases of athletes with congenital LVD localized in atypical site: LV basal inferior wall. Congenital left ventricular diverticula are often asymptomatic but may also cause life-threatening complications, sometimes as the first clinical presentation. Diagnosis is usually made after exclusion of acquired causes. The current case series highlights the relevance of risk stratification for cardiac events and the importance of a multimodal imaging approach, to detect location, extent, and morphology. The cases also emphasize the importance of individualized and tailored treatment strategy on the basis of clinical presentation, accompanying abnormalities and possible complications. Prospective long-term follow-up studies comparing different clinical care options, possibly in athletes as well, are needed to better understand the LVA/LVD evolution and to enable the creation of dedicated management guidelines.

## Supplementary Material

ytae036_Supplementary_Data

## Data Availability

The data underlying this article will be shared on reasonable request to the corresponding author.

## Lead author biography

Dr Giuseppe di Gioia is a medical doctor, cardiologist specialized in Sport Cardiology. Since 2016, he has been working with National, International and Olympic Athletes at the Italian National Olympic Committee (Institute of Sport Medicine and Science of CONI in Rome) as a member of Italian Olympic medical team. He is currently actively involved in cardiovascular research related to high-level athletes, conducting studies on relevant topics such as athlete’s heart adaptations to training, hypertrophic cardiomyopathy, arrhythmic disease, lipid disorders in athletes, and the prevention of sudden cardiac death.

## Supplementary material

**Consent:** The authors confirm that written consent for submission and publication of these case reports including images and associated text has been obtained from the patients in accordance with COPE guidelines (patients signed written informed consent).

**Funding:** This research did not receive any specific grant from funding agencies in the public, commercial, or non-profit sectors.
